# Philadelphia Hospital

**Published:** 1839-05-11

**Authors:** W. W. Gerhard


### MEDICAL EXAMINER.

DEVOTED TO MEDICINE, SURGERY, AND THE COLLATERAL SCIENCES.

No. 19.] PHILADELPHIA, SATURDAY, MAY 11, 1839. [Vol. II.

### CLINICAL LECTURE.

### PHILADELPHIA HOSPITAL.

### LECTURE ON PNUEMONIA—(Continued.)

By W. W. Gerhard, M.D.

In my last lecture I related to you a case of pneumonia complicated with disease of the brain and of the internal membrane of the heart; but as the former lesion was the immediate cause of death, you may readily understand that the symptoms connected with the cardiac affection must have been, to a great degree, obscured. These double complications are more frequently met with than any others in pneumonia, and are much more difficult to distinguish amidst the complicated symptoms of this form of the disease. It is well, therefore, from time to time to examine such cases as are met with at long intervals, and present these complications in a more simple form. These are too rare for us to witness during the ordinary duration of a course of lectures; but from time to time we observe very interesting scattered examples of the kind, which we can recall by looking over our notes, and then compare them with such cases as may chance to present themselves at the time. As an illustration of this form, I will relate the following case:

A labourer, of stout, muscular frame, twenty-eight years of age, was admitted on the 11th of August, 1838. He is an Irishman; has been seven years in America, employed at various railroads and canals; drinks spirits freely when at work, and has never been seriously ill, except from a scald last June. He had an attack of intermittent in the year 1833, and then had a slight cough, with hæmoptysis. He is subject to epistaxis. In other respects, has enjoyed vigorous health.

On the 2d of August was at work in the hold of a vessel, which was intensely hot; (thermometer in the shade, was, in the open air, at 97°;) there was much bilge water in the hold, and the patient had drunk largely of cold water from a neighbouring pump, although he was accustomed to drink water of the temperature of the air.

On the 3d was taken with pains in the whole right axilla, and cough, which was checked, as it were, by the pain. Remained in bed part of the day, then attempted to rise for a short time, but has kept his bed ever since. He had a short, dry cough, from the beginning, and thick, viscid expectoration, which soon became of a reddish tint. His mouth was dry, with great thirst, nausea, but no vomiting; no distinct chill.

His treatment was confined to a single bleeding the day before his entrance.

On the 11th he was extremely oppressed with severe pain in the right axilla, cough, and pneumonic sputa. He was cupped on the axilla, and took calomel gr. $\frac{1}{3}$, and pulv. Dover. gr. iv., every two hours.

On the 12th I saw him for the first time, and then he presented the following symptoms:

Since yesterday evening has taken the powders every two hours; feels better, can cough more easily; flushed face, dark, livid redness; a little stupor, but intelligence correct, and memory passably good; no cephalalgia; no ringing in ears, but some vertigo on rising; skin rather dry, except at forehead. (Sweat very slight, and only on taking warm drinks.) Tongue coated with white fur, dry and red generally. Cough short, suppressed. Expectoration small in quantity, of a rusty tint, and very viscid. Pulse 125, full, moderately resisting. Respiration 38, high, noisy. Had eight stools since entrance. Continue powders; diet.

13*th.*—Less dull, brighter intelligence; no cerebral expression, except slight dullness. Nostrils dilating forcibly; face flushed, of a dark red tint. Tongue dry, chapped, a little coated. Appetite better; four or five stools in the night. Cough short and rare. Expectoration still rusty and viscid, mixed with a thinner liquid. Decubitus dorsal; skin dry and hot; abdomen a little tympanitic, not tender since the powders; (had a slight dysentery at night;) pulse 114, full, resisting; respiration 36, high; percussion dull on right, resounding clearer towards summit; at same part, respiration extremely bronchial, and very distinct bronchophony; (a grating sound from friction of the pleura is heard at the middle of the lung;) the bronchial respiration is mingled with fine crepitus on coughing; vesicular murmur at base, in axilla, very bronchial; fine crepitus after cough, and bronchial respiration; posteriorly, bronchial respiration at the summit, with bronchophony; bronchial respiration and crepitus at root; intense bronchophony continues nearly to back, where there is abundant subcrepitant rhoncus; left side, respiration puerile throughout; no rhoncus; percussion, posteriorly, flat in right side, clear in left.

Impulse of heart greater than natural; first sound loud; second, nearly natural; impulse perceived on the right side more distinctly than left; slight dilatation of right side of chest.

Substitute for calomel and pulv. Dover.,

℞.—Mass. ex Hyd. gr. ij.
Carb. Ammon. gr. iij.

Venesection p. r. n. Foot bath t. in die.

14*th*—*Ptisans.*—After bleeding, was relieved; less dyspnœa. This morning, at sunrise, the dyspnœa was much increased, and mucous rhonchus was abundant in the lungs. He then took

Carb. Ammon. gr. v., and Anod. Liq. Hoffman, ʒss., q. ½ h. Pediluvium: sinapism to thorax.

At 11½, dyspnœa; anxious countenance, but less severe; bluish tint of lips and face; mucous rhonchus heard at distance; cough loose; expectoration thinner, of a light prune juice colour. Intelligence quite clear. Pulse 130, full and compressible. Respiration 48, high.

15*th.*—*Autopsy.* Exterior stout, muscular; ecchymosis over large portion of trunk and limbs; rigidity.

*Thorax.*—Percussion flat in whole right axilla and anterior portion of same side; dilatation in axilla evident; the right lung filled up nearly whole of that side of the chest; a few ounces of liquid were found on its anterior margin; the lung covered by layer of false membrane, yellow, soft, easily detached, about thickness of writing paper, forming a complete coat for lung; most uniform and thick on diaphragmatic portion; pleura brightly injected; in portions of its extent, membrane not yet complete, presenting a ceribriform appearance. At the base of lung, the pleura is evidently thickened after the false membrane is detached; opaque and very firm. The vessels are seen ramifying the pleura when detached from lung. Upper lobe perfectly solid throughout; immensely hypertrophied; three inches longer than upper lobe of left side, one inch thicker, one and a half inches broader; when cut into, found to be infiltrated with pus throughout, flowing from it abundantly after simple incision. At upper one-fifth the puriform conversion is perfect, though the granular aspect is preserved; the yellowness is of uniform intense colour; in the rest of its extent the degree of purulent infiltration varies; nearly perfect in about one-fourth extent of lung, chiefly anteriorly; elsewhere, intense red colour, mottled with yellow; some granulations becoming yellow, while intervening substance is still red; the cellular substance intervening being still red. No cavity in the lobe; bronchial tubes are full of a purulent liquid, of an intense red colour. They are opaque, red, and yellow; the smaller tubes in the lobe are obliterated. From some of them, tubes of false membrane can be drawn out; about a one-sixth portion of this lobe permeable to the air. Lower lobe is infiltrated with a reddish liquid; granulated red, shaded with yellow. Bronchial tubes intensely red, less obstructed than in upper lobe, but the smaller tubes contain the same shreds of false membrane. Middle lobe contains air; is reddened, infiltrated with serum, and offers a few traces of granulations.

*Left lung.*—Upper lobe, soft; contains air; filled merely with reddish serum, adherent at lower margin; bronchial tubes filled with serum, reddened, but less than in right lung. Lower lobe is much contracted; bound down by cellular adhesions; tissue condensed, but permeable. Bronchial tubes large, but short; mucous membrane opaque, and reddened throughout.

Pericardium contains an ounce of serum, transparent.

*Heart.*—Right side, distended with large coagula, black, mixed with fibrin; large coagula in left side, half as large as in the right. It adheres very closely to columnæ carneæ, and presents dots of redness, apparently the commencement of organization. The semilunar valves of aorta much reddened, bright arterial colour; patches of false membrane over large portion of their extent; it is this forms a delicate pellicle, readily removed by handle of scalpel, and leaving the membrane itself smooth and shining. Between the laminæ of the valves are points of white cartilaginous substance. One of these valves then forms several orifices closed by the inner membrane. Mitral valve reddened, but less than aorta; presents no false membrane, and is scarcely thickened. A few traces of false membrane are found on internal membrane of ventricle near aorta. Semilunar valves of pulmonary artery are thin; no false membrane of the same tint as adjoining tissue of heart, while the semilunar valves of aorta are of much redder tint; firm, well organized, fibrinous coagula extended into pulmonary artery, thence into lung; very resisting, elastic; tough as half tanned leather. The aorta is filled with liquid blood; is reddened before the arch in thorax, below of a light orange tint; internal membrane thickened; cartilaginous spots easily detached. The redness of aorta diminishes in descending, and ceases at its bifurcation.

The other viscera were examined with care, but presented no important lesions.

This case is an interesting example of this form of disease, and shows you how very latent the secondary inflammation of the heart becomes in cases of pneumonia. The only symptoms were the increased impulsion, and the slight exaggeration of the second sound of the heart. It was not easy to examine the patient at different periods of the disease, and the state of the heart was not ascertained within the last day or two of life. You will generally find that the signs of secondary endocarditis are obscure, and are limited to a slight confusion in the character and loudness of the sounds. The dyspnœa is, in these cases, usually disproportionate to the extent of the pulmonary inflammation, and may place us upon the right path for discovering the new inflammation.

There was nothing unusual in the earlier symptoms of the pneumonia, which, at first, was extremely favourable in its character, until it suddenly assumed an aspect of greater danger from the supervention of the disease of the heart.

In these cases there is nothing peculiar in the treatment. When the inflammation of the heart occurs very early, blood-letting should be prescribed, perhaps, with greater freedom than in ordinary cases; it requires, also, the use of abundant means of local depletion, and of extensive counter-irritants upon the thorax and extremities. The calomel and Dover's powders are a most useful remedy in the treatment of asthenic pneumonia, and of the ordinary form of the disease after it has reached the third stage; but, in the present case, it was directed at an earlier period of the disease than that at which I am myself in the habit of prescribing it.

